# Otorhinolaryngological presentations of mucormycosis amid COVID-19 pandemic in a tertiary care hospital

**DOI:** 10.12669/pjms.38.8.6431

**Published:** 2022

**Authors:** Fazal-I-Wahid, Muhammad Saleem, Habib-ur-Rehman, Israr ud din

**Affiliations:** 1 Dr. Fazal-I-Wahid, FCPS, Department of Ear, Nose and Throat (ENT), Head and Neck Surgery, Medical Teaching Institute (MTI), Lady Reading Hospital (LRH), Peshawar, Khyberpakhtunkhwa (KPK), Pakistan; 2 Dr. Muhammad Saleem, FCPS. KMU Institute of Medical and Dental Sciences, Kohat, Pakistan; 3 Dr. Habib-ur-Rehman, FCPS, Department of Ear, Nose and Throat (ENT), Head and Neck Surgery, Medical Teaching Institute (MTI), Lady Reading Hospital (LRH), Peshawar, Khyberpakhtunkhwa (KPK), Pakistan; 4 Israr ud din, FCPS. Department of Ear, Nose and Throat (ENT), Head and Neck Surgery, Medical Teaching Institute (MTI), Khyber Teaching Hospital (KTH), Peshawar, Khyberpakhtunkhwa (KPK), Pakistan

**Keywords:** Mucormycosis, Co-morbidities, Rhino-orbito-cerebral mucormycosis, COVID-19 infection

## Abstract

**Objective:**

To describe presentations, comorbidities, investigations, and surgical treatment of patients with mucormycosis amid the COVID-19 pandemic in a tertiary care hospital in Khyber Pakhtunkhwa, Pakistan.

**Methods:**

This descriptive study was conducted at the department of ENT, and Head and Neck Surgery, Medical Teaching Institute, Lady Reading Hospital, Peshawar, Pakistan from June 2020 to June 2021. All the patients fulfilling the inclusion criteria were included. Patients with COVID-19 were diagnosed based on real-time polymerase chain reaction (RT-PCR). After diagnosing mucormycosis computed tomography (CT) scan and/or magnetic resonance imaging (MRI) were performed for subsequent surgical clearance. After taking informed consent demographic data were collected on a proforma and analyzed using SPPS version 25.

**Results:**

Out of 23 patients males were 14(60.9%), females were 9 (39.1%) with a male: female ratio of 1.5:1. Mean ± SD age was 51.26 ± 1.41 years. Nasal obstruction and headache were the most common (8, 34.8%) presentations. The commonest co-morbidities were hypertension with diabetes mellitus (8, 34.8%). Out of 23 patients, 16(69.6%) had COVID-19 PCR positive. The majority of patients (17, 73.9%) were not vaccinated against COVID-19. Most of the patients (9, 39.1%) had HbA1c levels of 7% to 8.9%. The commonest surgery was endoscopic debridement of paranasal sinuses (9, 39.1%), while the commonest CT scan finding was a heterogeneous lesion involving the nose, maxillary and ethmoid sinuses (12, 52.2%).

**Conclusion:**

Mucormycosis of paranasal sinuses with/or without intracranial extension is frequently seen in unvaccinated patients having uncontrolled diabetes and COVID-19 infection.

## INTRODUCTION

Mucormycosis is an infection caused by molds of mucor that is commonly present in the soil, decaying vegetables and fruits, air, plants, and sometime in the mucus secretions of human beings. The human body’s closed cavities like sinuses, lungs, and brain are commonly affected by this infection. People with uncontrolled diabetes and immunodeficient status are more proven to get mucor.^1^ In December 2019 a novel virus as Severe Acute Respiratory Syndrome Coronavirus 2(SARS- Co V-2) emerged in Wuhan city of China. Later on, WHO (World Health Organization) named this viral infection as COVID -19(Coronavirus Disease 2019). COVID-19 resulted in a pandemic because 206 million people were infected and almost 4.3 million died across the globe.^2^ The incidence of mucormycosis was previously mentioned as 0.005 to 1.7 per million populations and in diabetics, it was 0.14/1000 diabetics patients.^1^

The incidence of oto-rhino-laryngological involvement by mucormycosis in COVID-19 patients is yet to be discovered. However, case reports of mucor in COVID-19 patients are on the rise.^3^Luckily mucormycosis is not that alarming in Pakistan as compared to the neighboring country India. Data is not available in Pakistan on mucormycosis. However it is thought if sufficient preventive measures are not taken on an urgent basis, this condition may become a real threat as the condition is on the rampant rise in developing countries.^4^ Mucormycosis, the black fungus is not a contagious disease, but it had a great affinity for patients with immunocompromised status like uncontrolled diabetics, HIV infected patients, patients with organ transplants, and patients with cancer.^5^Patients with COVID-19 presenting with mucormycosis involving the orbit, sinonasal mucosa, and oral cavity present to the otorhinolaryngologist. The severity of symptoms depends upon the immune state of the patients, co-morbidities, duration of disease, and efficacy of treatment.^6^

There is a lack of study on otolaryngological presentations of mucormycosis in our province. Presentations of patients with mucormycosis to the otorhinolaryngological department are not infrequent, thus this study aimed to describe the presentations and management of patients with mucormycosis.

## METHODS

This was a descriptive prospective study conducted at the Department of Ear, Nose, Throat (ENT), and Head and Neck Surgery, Medical Teaching Institute (MTI), Lady Reading Hospital (LRH), Peshawar, Khyber Pakhtunkhwa (KPK), Pakistan from June 2020 to June 2021. During this study time, there were 2^nd^, 3rd, and 4^th^ waves of rampant rise in COVID -19 in our country. Although the elective services were stopped in our department, the patients with mucor were arriving on an emergency basis. All the patients fulfilling inclusion criteria and consenting to study were included, while the cases with disseminated mucormycosis beyond the head and neck region, those with shortened life expectancy due to advanced age, and those not willing for surgical debridement were excluded. All the patients were either clinically or histopathologically diagnosed cases of mucormycosis with a contemporaneous or previous history of COVID-19 infections.

Patients with COVID-19 were diagnosed based on real-time polymerase chain reaction (RT-PCR) of the specimen obtained from the nasopharynx or oropharynx. The diagnosis of mucormycosis in clinically suspected cases was based on the presence of fungal hyphae with peculiar characteristics of Mucorales fungi under microscopic examination from scraping prepared in 10% potassium hydroxide (KOH), and the diagnosis of mucormycosis was confirmed by microbiological culture or the diagnostic histopathological features of the biopsy. After diagnosing mucormycosis computed tomography (CT) scan and/or magnetic resonance imaging (MRI) of paranasal sinuses, orbit, skull base, and brain were carried out to determine the extent of disease for subsequent surgical clearance. The patients were referred from other wards either for taking a biopsy to confirm mucormycosis and /or surgical debridement of mucormycosis. After taking an opinion from an anesthesiologist regarding fitness for general anesthesia, patients were subjected to endoscopic and /or open debridement of mucormycosis. In cases the patient was not fit for general anesthesia biopsy was taken under local anesthesia from the lesion for tissue diagnosis.

After taking well-informed consent from each patient demographic data like clinical presentations, investigations, co-morbidities, radiological findings, COVID-19 Vaccinations, and surgical procedures were collected on a predesigned proforma. All the patients were treated medically by a physician for COVID -19 and mucormycosis. The surgical debridement included endoscopic ethmoidectomy and /or sphenoidotomy and /or frontal sinusotomy, endoscopic medial maxillectomy, clearance from orbits, and pterygopalatine fossa exploration for debridement of necrotic tissues. An open surgical approach was chosen for the involvement of the palate and skin of the face. Quantitative data were calculated as mean and standard deviation (SD), and qualitative data were denoted as frequency and percentage. The confidence interval was taken at 95% and p<0.05 was considered statistically significant.

## RESULTS

Out of 23 patients, males were 14(60.9%), females were 9(39.1%) with a male: female ratio of 1.5:1. All patients were in the age range 20 – 90 years with mean ± SD age 51.26 ± 1.41years. The main bulk of the patients was in the 4^th^ and 5^th^ decade of life (13, 56.5%) (Table-I). The majority of patients (11, 47.8%) presented to the ENT department from Acute Medical Unit, followed by COVID Complex (5, 21.7%). Nasal obstruction and headache were the most common (8, 34.8%) presentations, followed by a nasal obstruction solely by seven patients (30.4%). Among the co-morbidities, hypertension with diabetes mellitus were prevailing (8, 34.8%), followed by hypertension and diabetes mellitus separately at 21.7% and17.4% respectively. Out of 23 patients, 16(69.6%) had COVID-19 PCR positive. The majority of patients (17, 73.9%) were not vaccinated against COVID-19. Regarding diabetes mellitus status, most of these patients (9, 39.1%) had HbA1c levels of 7% to 8.9%.

**Table-I:** Age of patients in years (n-23).

Age Groups (Years)	Frequency	Percent
A (≤ 20)	0	0
B (21-40)	5	21.7
C (41-60)	13	56.5
D (61-8))	4	17.4
E (≥ 81)	1	4.3

Total	23	100.0

**Fig.1:**
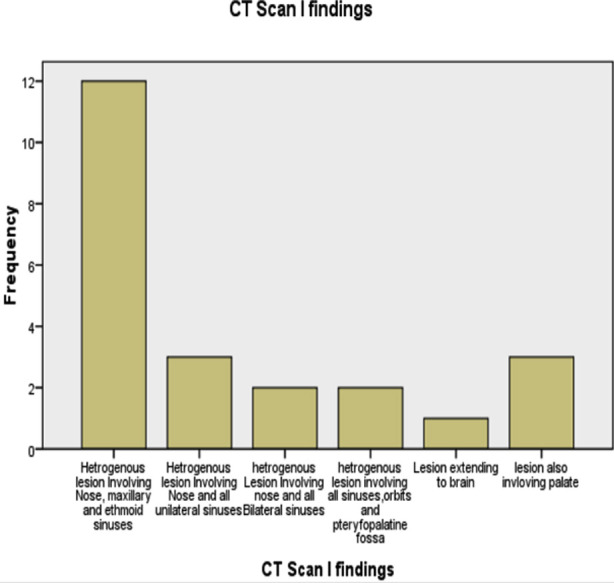
CT scan findings of patients with mucormycosis (n-23).

The commonest surgical procedure performed was endoscopic removal of lesions from the left side nasal cavity, maxillary and ethmoid sinuses (9, 39.1%)(Table-II). The commonest CT scan finding was a heterogeneous lesion involving the nose, maxillary and ethmoid sinuses (12, 52.2%), followed by a heterogeneous lesion involving unilateral maxillary, ethmoid, frontal and sphenoid sinuses (3, 13.0%) (Fig.1)

**Table II:** Demographics of patients (n-23).

Item	Frequency	Percent
***Source of admission***		
Acute Medical Unit (AMU)	11	47.8
COVID-19 Complex	5	21.7
Medical Departments	3	13.0
Pulmonology Department	3	13.0
Nephrology Department	1	4.3
Total	23	100.0
***Presentations***		
Nasal Obstruction	7	30.4
N.O +Headache	8	34.8
N. O+HD +Reduced Vision	3	13.0
N. O+HD +RV+ Visual Loss	2	8.7
Cheek Swelling and ptosis	2	8.7
Lesion on Palate	1	4.3
Total	23	100.0
***Co-morbidities***		
HTN+DM	8	34.8
HTN	5	21.7
DM	4	17.4
COPD	3	13.0
CRF	2	8.7
Malignancy	1	4.3
Total	23	100.0
***COVID-19 PCR Test***		
COVID-19 PCR Positive	16	69.6
COVID-19 PCR Negative	7	30.4
Total	23	100.0
***COVID-19 Vaccination Status***		
COVID -19 Vaccination yes	6	26.1
COVID -19 Vaccination No	17	73.9
Total	23	100.0
***HBA1c Level***		
HbA1c Level 5.5% - 6.9%	4	17.4
HbA1c Level 7% - 8.9%	9	39.1
HbA1c Level 9 % - 10 %	7	30.4
HbA1c Level > 10 %	3	13.0
Total	23	100.0
***Surgical procedures performed***		
Endoscopic removal of lesion from nose, maxillary and ethmoid sinuses Left side	9	39.1
Endoscopic removal of lesion from nose, maxillary and ethmoid sinuses Right side	6	26.1
Endoscopic clearance of lesion from orbits, pterygopalatine fossa	2	8.7
Debridement of involved Palate and Skin	1	4.3
Incision Biopsy from nasal mass	2	8.7
Full House FESS	3	13.0
Total	23	100.0

NB. NO= Nasal Obstruction, HD= Headache, RV= Reduced Vision, HTN=Hypertension,

DM = Diabetes mellitus, COPD= Chronic obstructive pulmonary disease,

CRF= Chronic renal failure FESS=Functional Endoscopic Sinus Surgery.

## DISCUSSION

Mucormycosis (Zygomycosis) is an uncommon lethal fungal infection caused by molds of mucormycetes. It is also known as black fungus, which is a fatal infection. Rhizopus and mucor are the commonest fungal genera leading to mucormycosis infection. Decaying food, soil, manure, and dust are the areas where Mucorales are existing frequently. Mucormycosis was first identified in 1855. The route of transmission of mucormycosis is through fungal spore inhalation, eating fungal-contaminated food, and infecting skin wounds with fungus. Immunocompromised patients and patients with uncontrolled comorbidities are the main victims of mucormycosis. Besides clinical manifestations, examination of fungal biopsy specimens and CT scan findings are helpful in its diagnosis. The most commonly occurring fungal infection is rhino-orbital cerebral mucormycosis.

Mucormycosis is reported at 34% in Europe, 31% in Asia, 28% in America, and 3% in each Australia and New Zealand.^7^Mucormycosis a fatal disease that results from the involvement of blood vasculature by a fungus which leads to mycotic thrombosis, ischemia, and necrosis of the involved tissues. The worldwide incidence of mucormycosis is reported in the range of 0.005 to 1.7 per million populations. The exact incidence of this disease is not known in Pakistan; hence this study was conducted.^8^In the current study males were predominant (60%) with mean ± SD age of 51.26 ± 1.41 years, which is in par with the results of Patel from India where 74.6% were men and mean age of the study population was 53.4 years (SD 17.1 years).^9^ Our study is also supported by Arora and colleagues, who found the mean age of the patient, 51.9 years, and male preponderance (66%).^10^Similar findings are also observed by Ramphul and colleagues.^11^ Thepredilection for males can be assumed due to more exposure of males to fungal spores based on prolonged out-doors engagement and oestrogenic protection.

The commonest symptoms of patients were nasal obstruction and headache (8, 34.8%), and nasal obstruction solely in seven patients (30.4%) in this study, which is contradictory to Arora’s result, which revealed that the most common symptoms were facial pain (35.58%), facial swelling (12.20%) and nasal blockage (28.3%).^10^ In the current study the commonest comorbidities were hypertension with diabetes mellitus (34.8%), followed by hypertension (21.7%) and diabetes mellitus (17.4%). Similarly, Osibogun also found that the most common co-morbidities were hypertension (74.2%), diabetes (30.3%), and asthma (10.2%).^12^ The reason may be uncontrolled hypertension and diabetes mellitus and injudicious prolonged use of steroids for COVID-19 treatment leading to mucor infection. We observed that out of 23 patients 16(69.6%) had COVID-19 PCR positive tests, which is simulating to study of Fouad, where RT-PCR was positive in 50% of patients.^13^

In this study majority of patients (17, 73.9%) were unvaccinated, which is coinciding with Arora’s study from India where 53 patients (88.3%) were non-vaccinated and only 11.7% of patients received vaccination against COVID-19.^10^Similarly Selarka also reported that majority of subjects (31, 66.0%) were not vaccinated for a COVID-19.^8^The probable explanation for lower vaccination rate is low literacy level, constrained government resources and false socio-cultural belief of public against vaccination in developing countries like Indo-Pak. In the present study majority of patients (9, 39.1%) had HbA1c levels between 7% and 8.9%, which is comparable to the literature with a reported mean value of HbA1c of 10.0 ± 2.1%.^14^,^15^

Surgical treatment of mucormycosis is the removal of involved necrotic tissues and fungal load followed by drainage and irrigation of paranasal sinuses. The types of surgery for mucormycosis depend upon the extent of the disease. We removed the lesion endoscopically from the nose and paranasal sinuses on the left and right sides in 9(39.1%) and 6(26.1%) patients respectively, which is comparable to the Selarka study, who performed modified Denker’s approach in 19(40.4%) patients and FESS in 19(40.4%) patients.^8^Likewise Shamanna also performed complete endoscopic debridement from the nose and paranasal sinuses only in 16 (80%) patients and endoscopic debridement from the nose and paranasal sinuses with orbital exenteration in 4 (20%) patients.^16^The CT scan finding in this study was heterogeneous lesion involving the nose, maxillary and ethmoid sinuses in 12(52.2%) patients, followed by a heterogeneous lesion involving unilateral maxillary, ethmoid, frontal and sphenoid sinuses in three (13.0%) patients. A similar study was conducted by Shamanna, and he found that a heterogeneous soft tissue density was observed in the nose and paranasal sinuses in 14(70%) patients; while in the Selarka study involvement of all sinuses by mucormycosis was found in 45(95.7%) patients.^8^,^16^

### Limitation:

The limitation of this study is a small sample size, so a large sample size with prolonged follow-up may be conducted to get in-depth knowledge of demographics and surgical outcomes of mucormycosis.

## CONCLUSION

Mucormycosis is a fatal fungal infection that can involve any part of the body of immunocompromised patients, especially with co-morbidities. Mucormycosis involving paranasal sinuses with/or without extension to orbit and cerebrum is frequently seen in unvaccinated patients with COVID-19 infection and uncontrolled diabetes. Besides medical treatment, surgical debridement of involved tissues and fungal load can minimize the mortality and morbidity of COVID-19 patients harboring mucormycosis.

### Authors’ Contribution:

**FIW** conceived and designed the study, is responsible for integrity of research.

**MS** did data collection. **HUR** Preparation of manuscript and critical review.

**ID** Data analysis and final approval of the version to be published.
